# TSPO: an emerging role in appetite for a therapeutically promising biomarker

**DOI:** 10.1098/rsob.210173

**Published:** 2021-08-04

**Authors:** Joshua Wang, Kate Beecher

**Affiliations:** Addiction Neuroscience and Obesity Laboratory, School of Clinical Sciences, Faculty of Health, Translational Research Institute, Queensland University of Technology, Brisbane, Queensland, Australia

**Keywords:** TSPO, obesity, appetite, RIM-BP1, neurosteroids, tanycytes

## Abstract

There is accumulating evidence that an obesogenic Western diet causes neuroinflammatory damage to the brain, which then promotes further appetitive behaviour. Neuroinflammation has been extensively studied by analysing the translocator protein of 18 kDa (TSPO), a protein that is upregulated in the inflamed brain following a damaging stimulus. As a result, there is a rich supply of TSPO-specific agonists, antagonists and positron emission tomography ligands. One TSPO ligand, etifoxine, is also currently used clinically for the treatment of anxiety with a minimal side-effect profile. Despite the neuroinflammatory pathogenesis of diet-induced obesity, and the translational potential of targeting TSPO, there is sparse literature characterizing the effect of TSPO on appetite. Therefore, in this review, the influence of TSPO on appetite is discussed. Three putative mechanisms for TSPO's appetite-modulatory effect are then characterized: the TSPO–allopregnanolone–GABA_A_R signalling axis, glucosensing in tanycytes and association with the synaptic protein RIM-BP1. We highlight that, in addition to its plethora of functions, TSPO is a regulator of appetite. This review ultimately suggests that the appetite-modulating function of TSPO should be further explored due to its potential therapeutic promise.

## Graphical abstract

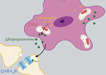

## 1. Introduction

An inflamed brain is an emerging phenotype of obesity [1,2]. Specifically, neuroinflammation at the hypothalamus has been shown to dampen anorexigenic neuronal activity and promote orexigenic neuronal activity [3,4]. This foundational finding has implicated neuroinflammation as a cause of appetite dysfunction, and subsequently, diet-induced obesity. In mimicking the obesogenic Western diet, rodent studies in which animals were fed a high-fat [3], high-sugar [5–7] or combination high-fat high-sugar [8] diet precipitated hypothalamic neuroinflammation. The cellular mechanisms of diet-induced inflammation are currently being investigated, with microglia [9–13] and astrocytes [14,15] adopting pro-inflammatory states in response to overnutrition.

The translocator protein of 18 kDa (TSPO) is a modulator of neuroinflammation [16–19] that may also regulate diet-induced obesity [20]. TSPO is located predominately within the outer mitochondrial membrane [21] in various tissues where it regulates a plethora of processes, including steroidogenesis [22], mitochondrial energetics [23], porphyrin synthesis [24,25] and apoptosis [26]. TSPO is also expressed in non-diseased human and wild-type rodent brains in astrocytes, microglia and neurons in a region-dependent manner [27–29]. TSPO is expressed sparsely throughout the brain, but is concentrated at the olfactory bulb, the choroid plexus, the cerebellum and the ependyma (reviewed in [27]). However, the regional and cellular expression of TSPO is altered across a variety of disease states [30]. TSPO is most well known as a marker of neuroinflammation as its expression is constitutively low but greatly increased in pro-inflammatory microglia [31–33]. Consequently, an array of TSPO ligands have been developed for positron emission tomography (PET) imaging, allowing for *in vivo* human data on neuroinflammation to be generated [34]. As a result, TSPO has a variety of highly specific ligands [35–37] available to dissect its function, as well as a clinically used agonist that is anxiolytic: etifoxine [38]. Therefore, TSPO may represent a therapeutically viable target for the treatment of diet-induced obesity. Although it has been implicated in feeding previously [39,40], how TSPO regulates appetite in the brain has not been explicitly explored. This review discusses three putative mechanisms for how brain TSPO may alter appetitive circuits: the TSPO–allopregnanolone–GABA_A_R (TAG) axis, modulation of tanycytic glucosensing and altering RIM-BP1 function. By building on these foundational findings, future research may identify a strategy to target TSPO as an obesity treatment.

## 2. TSPO increases the efficiency of GABAergic transmission in appetitive circuits via the TAG axis

The channel-like appearance of TSPO, as well as the identification of an evolutionarily conserved cholesterol recognition amino acid consensus sequence [41], suggests that it is involved in cholesterol transport. Cholesterol import into the mitochondrial intermembrane space is the rate-limiting process in the synthesis of neurosteroids [42,43]: brain-specific steroids that are synthesized de novo within the brain [44,45]. Many studies demonstrate that TSPO ligands, including TSPO's endogenous ligands, the endozepines [46], influence the production of neurosteroids [45,47–50]. However, recent studies have shown that TSPO knockout models maintain neurosteroid synthesis [51–54], dismissing the long-held belief that TSPO knockout was lethal in rodent models [55]. TSPO is therefore not essential for cholesterol import, but heavily influences this process [56,57]. As a result, the original name ‘peripheral benzodiazepine receptor’ [58] is now largely replaced by TSPO to better reflect its hypothesized function in cholesterol transport [22].

**Figure 1.**
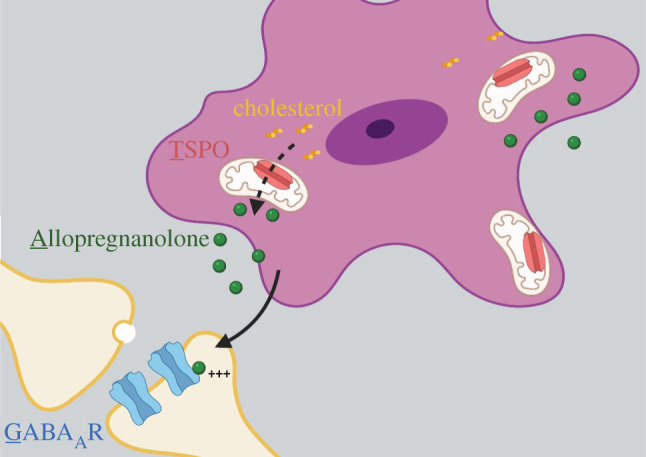
The TAG axis. Allopregnanolone synthesis begins with cholesterol's import into the mitochondrial matrix through TSPO. This cholesterol import is the rate-limiting step in allopregnanolone synthesis. As a hydrophobic molecule, allopregnanolone is then able to diffuse freely to surrounding cell types, including GABAergic synapses. When bound to GABA_A_Rs, allopregnanolone acts as a positive allosteric modulator, increasing the activity of these receptors. Figure produced using Biorender software.

The neurosteroids that TSPO assists in synthesizing are produced in both neurons and glia [59] throughout many brain regions [60]. The specific neurosteroids that TSPO produces are altered by different TSPO ligands and there is conflicting evidence to implicate TSPO in the production of specific neurosteroids [61]. However, allopregnanolone production has universally been found to be increased in the presence of a TSPO ligand, including PK11195 [18,62], etifoxine [63,64], FGIN1-27 [65,66], XBD-173 [63], YL-IPA08 [67] and CB-34 [62]. While traditional steroids act through nuclear receptors to alter transcriptional events, neurosteroids act independently of nuclear steroid hormone receptors [68,69]. Instead, neurosteroids bind to an allosteric site on ionotropic GABA_A_ receptors [70–73]. The most potent of these neurosteroids, allopregnanolone, acts as a positive allosteric modulator of GABA_A_Rs [74] and increases its effectiveness at producing inhibitory potentials. GABA_A_Rs are widely expressed throughout the brain and have been implicated in the regulation of appetite [75]. Therefore, it is not surprising that allopregnanolone is a major regulator of feeding [76]. Based on these findings, we hypothesize that TSPO influences sucrose overconsumption through a potential signalling axis composed of allopregnanolone and GABA_A_Rs as downstream effectors. This signalling cascade is termed the TAG axis (figure 1). Given that TSPO modulates the rate-limiting step in the production of allopregnanolone [42,43], alterations in TSPO expression or activity would hypothetically alter the overall activity of the TAG axis.

## 3. Tanycytic TSPO modulates glucosensing

**Figure 2.**
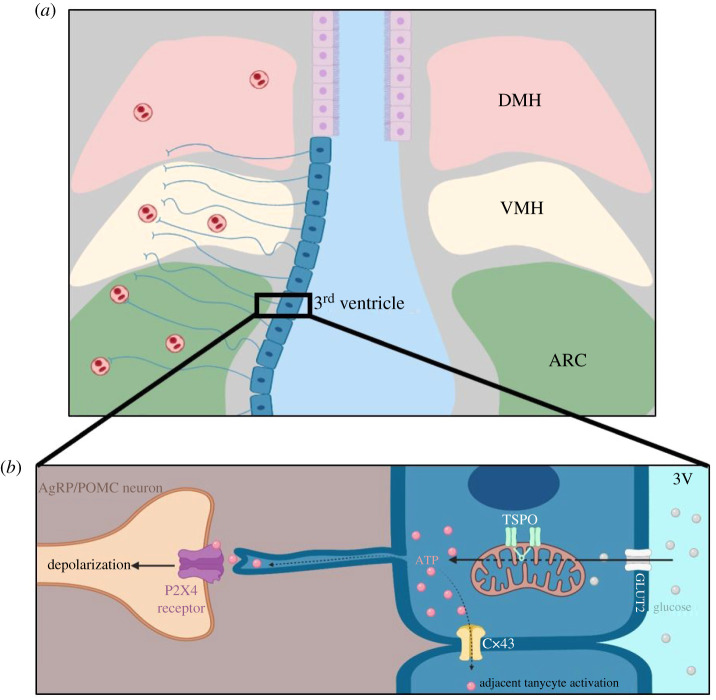
TSPO regulates glucosensing in tanycytes. (*a*) Schematic of the coronal section of the rodent brain. Tanycytes continuously line the ventral portion of the third ventricle (right-hand side tanycytes removed for clarity), and their basal projections infiltrate the surrounding dorsomedial hypothalamus (DMH), ventromedial hypothalamus (VMH) and arcuate nucleus (ARC). These projections contact both the hypothalamic parenchyma, as well as capillaries. (*b*) TSPO and tanycytic glucosensing. Glucose is present in the CSF at concentrations that reflect blood glucose levels. When the CSF passes the ventral portion of the third ventricles, it is transported into tanycytes via glucose transporter 2 (GLUT2). Then, glucose is oxidized in the mitochondria to produce ATP. This process is positively regulated by TSPO. The resultant ATP is then used to activate adjacent tanycytes by passing through connexin 43 hemichannels (Cx43). Tanycytic ATP is then also used to depolarize adjacent AgRP (orexigenic) and POMC (anorexigenic) neurons by binding to purinoceptors (predominately the P2X4 receptor), ultimately changing the appetitive state of the brain. Figure produced using Biorender software.

Tanycytes were first described as lengthened bipolar ependymal cells lining the ventral portion of the third ventricle [77]. Their name is derived from the Greek term *tanus*, meaning stretched/elongated [78]. Although there were transient hypotheses that tanycytes were a neuronal subpopulation [79], they are now considered radial glial-like cells that maintain stem cell phenotypes [80]. There are now promising foundational single-cell transcriptomic data available for tanycytes [81,82], which has resolved five transcriptionally unique tanycytic cell types [81]. Recently, transgenic labelling of tanycytes has confirmed a number of their structural properties [83]. Tanycytes consist of a proximal cell body lining the ventricular surface of the third ventricle, with a single distal process that traverses the arcuate nucleus, ventromedial nucleus and dorsomedial nucleus of the hypothalamus, contacting capillaries and the neural parenchyma (figure 2*a*). Tanycytes therefore represent a three-way physical connection with feeding-related hypothalamic circuits, the cerebrospinal fluid (CSF) and the circulation [84]. It is, therefore, not surprising that an emerging body of research now implicates tanycytes in appetite modulation [85]. Additionally, tanycytes have some of the highest expression of TSPO in the entire brain [27,39], likely due to the fact that TSPO is expressed highly in explanted neural stem cells [86].

Given that CSF contents are proportional to blood contents for many solutes such as glucose [87], the structure and location of tanycytes allow them to monitor metabolic cues from the CSF to subsequently signal these alterations to appetite-related neurons in the hypothalamus. Firstly, the ventricular surface of tanycytes are equipped with microvilli extending into the lumen of the third ventricle [88], allowing them to sense a variety of nutrient signals in the CSF such as glucose [89–91], artificial sweeteners [92], amino acids [93] and lipids [94,95], which ultimately influence the transcriptomic state of these cells [81,96]. Depending on nutritional status, tanycytes can regulate the blood–brain barrier and the permeability of CSF constituents [97]. For example, tanycytes release vascular endothelial growth factor (VEGF) under fasting conditions, resulting in an increased permeability of metabolic signals to hypothalamic neurons [98]. Overnutrition also alters tanycytic control over metabolic signalling; blocking leptin [99] and ghrelin [100] from reaching the hypothalamic parenchyma, where they would normally modulate hypothalamic neurons to alter the appetitive state [101,102]. Tanycytes also directly modulate these neurons by altering their expression of orexigenic and anorexigenic neuropeptides [39,91,103–106]. The role of tanycytes in appetite has been confirmed through many loss-of-function studies; tanycyte ablation [105,107,108] and genetic ablation of tanycytic glucosensing machinery [103,104,106] alter feeding and body weight.

As previously stated, TSPO is highly expressed in tanycytes [27,39]. Additionally, tanycytes respond to inflammatory signals by producing chemokines that modulate feeding, suggesting a possible role for TSPO in tanycyte-modulated appetite [109]. Recently, it was shown that genetic ablation of tanycytic TSPO decreases food consumption in mice fed a high-fat diet [39]. Additionally, administering the TSPO antagonist PK11195 into the third ventricle also decreases feeding in an identical animal model [39]. There is further evidence to suggest that TSPO is important for the function of tanycytes in metabolic homeostasis. For example, tanycytes secrete diazepam binding inhibitor (DBI)—the endogenous ligand for TSPO [110–112]. However, DBI is post-translationally cleaved into a number of endozepine products, some of which do not interact with TSPO [113]. It is therefore unknown at this stage how DBI or its TSPO-binding cleavage product triakontaheptaneuropeptide [114] mediate tanycyte function. However, there is emerging evidence that has led us to hypothesize that tanycytic TSPO regulates appetite by altering ATP production during glucosensing.

TSPO has a potential role in modulating how tanycyte glucosensing affects feeding neurons within the hypothalamus. Upon sensing glucose in the CSF, tanycytes are activated through an ATP wave [90] propagated by connexin-43 gap junctions between adjacent tanycytes [115]. Activated tanycytes also release ATP, which then diffuses into the arcuate nucleus, where it depolarizes both orexigenic and anorexigenic neuronal populations [116,117]. *In vivo*, tanycytic activation caused hyperphagia only in the fed state [116], demonstrating that aberrant tanycyte signalling can override appetite homeostasis. We predict that TSPO modulates the ATP-dependent activation of adjacent tanycytes and appetite-regulating neurons in the ARC. TSPO knockout in both human [118] and rodent [51,119] microglia decreases ATP production. On the other hand, ATP production increases during microglial TSPO overexpression [51] or during the administration of TSPO ligands [119]. In A2/29 cells, a tanycyte cell culture model, TSPO knockout increases ATP production, indicating a potentially unique role for TSPO in tanycyte energetics [39]. The mechanism for TSPO's regulation of ATP production is unknown; however, it has been recently hypothesized that this mechanism involves TSPO-mediated phosphorylation of F_1_F_O_-ATP synthase [23], given that TSPO ligand administration increases the extent of this phosphorylation [120]. Therefore, TSPO may modulate the ATP-dependent tanycyte response to CSF glucosensing, ultimately modulating the activation of the appetite-regulating Agouti-related peptide (AgRP)- and proopiomelanocortin (POMC)-expressing neurons in the arcuate nucleus (figure 2*b*).

## 4. TSPO interacts with RIM-BP1 to potentially influence feeding

TSPO may also influence appetite by altering synaptic plasticity. Galiègue *et al*. [121] discovered that a protein now identified as RIM-binding protein 1 (RIM-BP1) [122] binds to TSPO-specific motifs. Although RIM-BP1 is located at the pre-synaptic active zone [123], overexpression of TSPO causes an increase in RIM-BP1 protein expression in mitochondrial extracts [121]. These data suggest that TSPO interacts physically with RIM-BP1, modulating its subcellular localization. RIM-BP1, together with RIM-BP2, interact with voltage-gated calcium channels at the pre-synaptic terminal, which is an evolutionarily conserved function [123]. This interaction is believed to contribute to the positional priming of exocytic machinery [124,125], which ultimately increases the number of synaptic vesicles released [126]. Although the role of RIM-BP1 is not as critical in mammals [127] as *Drosophila* models [128,129], RIM-BP1 mutations and polymorphisms are correlated with autism in humans [130–132]. While RIM-BP1/RIM-BP2 double knockout mice show no difference to RIM-BP2 single KO mice [133], the effect of RIM-BP1 single knockout was not assessed in this study. A different RIM-BP1 single knockout study using mice showed decreased Ca^2+^ signalling in ribbon synapses [134]. Although RIM-BP1 is rarely studied in isolation, these studies show that decreased availability of RIM-BP1 at the cytomatrix active zone (CAZ) is not fully compensated for by RIM-BP2 in murine neurons, demonstrating that RIM-BP1 is essential for physiological brain function. Therefore, if TSPO interacts with RIM-BP1, it will probably precipitate a change in synaptic function.

RIM-BP1 is also implicated in appetite. Firstly, RIM-BP1 is highly expressed in brain regions associated with the limbic system [122], a key circuit in feeding-related reward [135]. RIM-BP1 antisense RNA (TSPOAP1-AS) hypermethylation at the promoter region is correlated with obesity and plasma cholesterol levels from a small human sample [136], suggesting a role for RIM-BP1 in obesity and steroid homeostasis. RIM-BP1's association with obesity, binding to TSPO and predominate expression in the limbic system suggest that RIM-BP1 may modulate TSPO's role in appetite regulation. However, no further binding studies (such as immunoprecipitation) have been published to confirm the interaction between TSPO and RIM-BP1. Given that in the healthy brain, RIM-BP1 is predominately expressed in neurons [137], and TSPO is predominately expressed in glia [30], it is difficult to test this putative mechanism. One potential strategy to address this is to use cultured olfactory bulb cells to test the interaction of RIM-BP1 and TSPO expression in the synaptic site of the olfactory neurons and the mitral cells, given that the olfactory bulb is one of the only confirmed sites with neuronal TSPO expression in the healthy brain [21,30].

## 5. Brain TSPO neuroplasticity in response to nutritional change

TSPO expression in the brain changes drastically in response to disease [30], potentially through a CpG methylation-mediated epigenetic mechanism [138]. It is therefore not surprising that TSPO expression is also sensitive to alterations in diet. Four studies, all conducted in the past decade, have documented alterations in TSPO as a result of diet (table 1). Interestingly, all studies assess the impact of overnutrition. These studies demonstrate an alteration in TSPO levels [139–141] and appetite-modulating function [39] due to overnutrition.

**Table 1.** Changes in brain TSPO due to overnutrition.

species	age	conditions	TSPO expression measurement method	regions assessed	TSPO change	regions with change	reference
human	range: 19–80 years mean ± s.d.: 53.2 ± 19.1 years		[^11^C]PBR28 PET	grey matter frontal cortex temporal cortex occipital cortex parietal cortex hippocampus thalamus	[11C]PBR28 PET signal (indicative of TSPO protein expression) negatively correlated with BMI	grey matter frontal cortex temporal cortex occipital cortex parietal cortex hippocampus thalamus	[139]
wistar rats (male)	5 weeks at beginning of the experiment	12 weeks of ad libitum standard chow + 5% sucrose solution provided in intermittent-access two-bottle choice drinking paradigm (24 day^−1^, 3 d wk^−1^)	[^18^F]DPA714 PET	cerebral cortex hippocampus thalamus caudate-putamen	increased PET signal, indicating increased TSPO protein expression	neocortex hippocampus thalamus caudate-putamen	[140]
C57/Bl6 mice (male)	15–17 weeks	ob/ob (leptin deficient) compared to wild-type	[^3^H]-PK11195 PET	coronal sections at the level of hypothalamus/hippocampus (stereotactic coordinates not specified)	increased PET signal, indicating increased TSPO protein expression	hippocampus choroid plexus of third ventricle	[141]
		standard diet					
			[^3^H]-PK11195 saturation binding on brain membrane extracts	whole brain	increased [^3^H]-PK11195 binding, indicating increased TSPO protein expression	whole brain	
	4–7 weeks at beginning of the experiment	HFD (4 weeks) compared to standard diet		tanycytes	genetic ablation of tanycytic TSPO decreased food intake and weight gain compared to wild-type in HFDmice; however, this ablation does not impact food intake or weight gain in standard diet mice	tanycytes	[39]
					ICV PK11195 resulted in no change in hypothalamic POMC expression of HFD mice, whereas the same treatment increased hypothalamic POMC expression in standard diet mice		

Undernutrition may also dysregulate TSPO signalling in the brain, given that anorexia nervosa is also considered to be driven by immune processes [142]. TSPO PET studies analysing patients with major depressive episodes [143] and serious self-harming behaviour [144] have included patients with anorexia nervosa. However, neither of these studies have had the statistical power necessary to analyse the effect of anorexia nervosa independently on TSPO expression. It is therefore still unknown if TSPO plays a role in conditions of depleted appetite, such as anorexia nervosa.

## 6. Conclusion

Neuroinflammation is emerging as a pathogenic process underlying a variety of diseases, including obesity. Fortunately, this has resulted in a combined effort in the neuroscience community to develop tools to effectively measure and control neuroinflammation [34]. This work has gifted the neuroinflammation biomarker TSPO with an evolved repertoire of tracers and ligands, instilling it with excellent translational potential. However, how TSPO regulates appetite in diet-induced neuroinflammatory obesity is not widely discussed. We have outlined three putative biochemical mechanisms through which TSPO modulate appetite. Various studies that have characterized TSPO changes due to overnutrition were also synthesized, demonstrating that brain TSPO is responsive to the metabolic environment of the individual. This analysis also demonstrated the lack of research exploring TSPO's potential involvement in anorexia nervosa. By exploring the biochemical mechanisms outlined, the neurological consequences of an obesogenic Western diet can be better characterized. Additionally, these explorations may allow for the repurposing of TSPO ligands as anti-obesity medication.

## Data accessibility

This article has no additional data.
